# Effects of Continuous Care Model Based Non-Pharmacological Intervention on Sleep Quality in Patients with Type 2 Diabetes Mellitus: A Randomized Controlled Clinical Trial

**Published:** 2015-04

**Authors:** Shahla Khosravan, Ali Alami, Somayyeh Golchin Rahni

**Affiliations:** 1 Department of Community and Mental Health, School of Nursing and Midwifery; Social Determinants of Health Research Centre, Gonabad University of Medical Sciences, Gonabad, Iran;; 2 Department of Health, School of Public Health; Social Determinants of Health Research Centre, Gonabad University of Medical Sciences, Gonabad, Iran;; 3 Department of Medical Surgical Nursing, Gonabad University of Medical Sciences, Gonabad, Iran

**Keywords:** Continuous Care Model, Non-pharmacologic Treatment, Pittsburgh Sleep Quality Index, Sleep Disorder, Type 2 Diabetes Mellitus

## Abstract

**Background:**

Sleep is an important aspect of healthy lifestyle. One of the prevalent Diabetes mellitus-related non-vascular complications is sleep problems. The aim of this study was to investigate the effects of a non-pharmacological care plan designed based on the Continuous Care Model (CCM) on sleep quality in patients with type II diabetes with two month follow up.

**Methods:**

This randomized controlled clinical trial was conducted from May to November 2012 among 68 the patients with type II DM referring to the Diabetes Clinic of Gonabad University of Medical Sciences. The study instrument consisted of a self-report demographic questionnaire and the Pittsburgh Sleep Quality Index. The gathered data were analyzed via SPSS (V. 20) using t-test and Chi-square statistics.

**Results:**

After the intervention, the study groups did not differ significantly in terms of sleep quality (0.628). However, the study findings revealed that the interventional group’s sleep quality improved significantly after the intervention (P<0.001).

**Conclusion:**

Non-pharmacologic intervention according to CCM improved the sleep quality in the experimental group. Sleep care is a matter of great importance in diabetes mellitus, which deserves particular attention. The present study adds to the growing literature of the use of non-pharmaceutics intervention to improve sleep disorders of diabetic patients.

**Trial Registration Number:** IRCT201202269140N1.

## Introduction

Diabetes Mellitus (DM) is a prevalent disease and a serious health problem worldwide. In 2013, 382 million people had diabetes; this figure is expected to rise to 592 million by 2035.^1^ The number of diabetic patients in Iran is more than three million people; this triples every fifteen years.^2^

DM causes many different acute and chronic complications that in turn result in most of the DM-related deaths. Chronic complications include vascular and non-vascular complications. Careful management of DM, careful maintenance of hemoglobin A_1C_ at normal range, and lifestyle modifications help prevent and treat vascular complications.^2^^,^^3^ One of the prevalent DM-related non-vascular complications is sleep problems. Adequate rest and sleep is an important aspect of healthy lifestyle. Diabetic patients, compared to healthy people, experience more insomnia, daily sleepiness,^4^ and obstructive sleep apnea.^5^ The prevalence of sleep problems in diabetic patients is 42–71%.^6^^,^^7^ Factors contributing to DM-related sleep problems include, but not limited to, obesity, sleep apnea, peripheral neuropathy-related pain and discomfort, and nocturnal hyperglycemia and hypoglycemia.^8^ On the other hand, sleep problems result in hormonal fluctuations, immunologic disorders, and metabolic abnormalities, which in turn aggravate the DM-related problems and cause fluctuations in blood glucose level.^9^ Studies have shown that sleep cycle significantly affects body metabolism. Accordingly, glucose metabolism decreases during the non-REM sleep and increases during wakefulness. Sleep deprivation and disturbed sleep patterns increase the activity of hypothalamic-pituitary-adrenal axis, which in turn increases the nocturnal level of blood cortisol and predisposes the patients to insulin resistance, glucose intolerance, and increased hunger.^1^^,^^10^ Moreover, during sleep, the level of leptin-an appetite-regulating peptide increases. Consequently, insomnia and sleep deprivation result in decreased level of leptin, increased level of cortisol, and decreased glucose intolerance.^11^

Despite the prevalence of DM-related sleep problems^6^^,^^7^^,^^12^ and the effects of sleep quality on glucose metabolism, energy balance, effective DM management,^13^ and also need to diagnosis and treatment of sleep disorders according to their exact type,^8^ diabetic patients receive little, if any, care regarding their sleep problems.^13^ Sleep problems are usually managed either pharmacologically or non-pharmacologically.^14^^,^^15^ Currently, pharmacologic interventions are used so much extensively that non-pharmacologic interventions have been largely forgotten. Most of the sleep specialists also prefer to prescribe tranquilizers and sedatives for the management of sleep problems. However, these agents have adverse effects, such as drug dependence and relapse of insomnia after discontinuation of treatment.^16^

Non-pharmacologic treatment of sleep problems includes interventions such as sleep hygiene education, relaxation, stress management, and cognitive therapy.^17^ The treatment option is selected according to the healthcare providers’ experiences and patients’ preferences.^14^^,^^15^^,^^18^ Healthcare providers, particularly nurses, need to assess their patients’ sleep quality and to adopt effective strategies to promote their sleep.^18^ Nabilnoted that nurses tend to manage sleep problems non-pharmacologically. Nursing care is usually provided by using different nursing models.^19^ Nursing models provide a set of guidelines for efficient delivery of nursing services.^20^ The Continuous Car Model (CCM) is one of the many nursing models developed by nurses. CCM is the continuous process of establishing an effective, long-term relationship between client, as the Continuous Care Agent, and nurse, as healthcare provider. The main objective of CCM is to develop a comprehensive care plan to improve the patients’ knowledge, attitude, and practice and to maintain the continuity of care. It also aims at helping the nurses promote the patients’ health, manage their diseases, and prevent complications. CCM consists of four stages including orientation, sensitization, control, and evaluation.^19^^-^^22^

Previous studies have investigated and proved the effectiveness of CCM in managing different health problems in different patient populations.^19^^-^^24^ Considering the importance of managing diabetic patients’ sleep problems^25^ as well as the importance of using non-pharmacological interventions for management of sleep problems and their complications, we conducted this study aiming at investigating the effects of continuous care model on sleep quality in patients with type 2 diabetes.

## Patients and Methods

**Figure 1:**
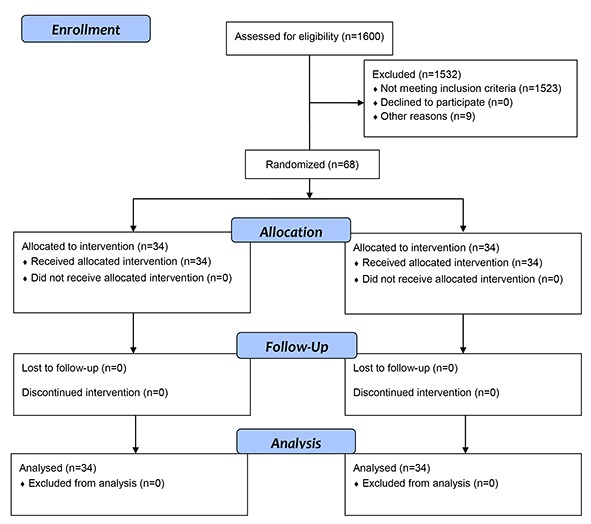
CONSORT flow diagram of the participants

This was a randomized controlled trial conducted from May to November 2012. The study population consisted of all patients with type II DM referring to the Diabetes Clinic of Gonabad University of Medical Sciences, Gonabad, Iran. The inclusion criteria were having 35–70 years of age, having a tendency to participate in the study, having the ability to fill in the study questionnaire, having no history of psychiatric disorders or amnesia, not being compelled to wake up repeatedly during night (for example to care for parents or to breastfeed baby), having no night working shift, and having no recent long-distance travel. Patients whose treatment or dietary regimen changed during the course of the study were excluded from the study. Initially, we administered the Pittsburgh Sleep Quality Index to all 1600 patients who met the inclusion criteria to identify patients suffering from sleep problems (Figure 1). The test results revealed that 97 patients had sleep problems. Then, a physician examined them, resulting in exclusion of twenty patients who had vision, hearing, or consciousness impairment or sleep-disturbance disorders. From the remaining 77 patients, we selected the required participants (68 patients) using simple random sampling by table-of-random-numbers. We then allocated 68 participants into the intervention and control groups using Balanced Block Randomization. There was a total of 6 blocks with 4 patients in each. In a fifteen-minute meeting, we then provided the study participants with information about the aim and the process of the study and invited them to read and sign the written consent form of the study.

Based on previous research^24^ and the researchers’ prediction to decline 30% sleep disorders after the trial in the intervention group, the required sample in each group was calculated as below.

$$n=\;\frac{2\;{\left(z_{1-\frac{\alpha }{2}}+z_{1-\beta }\right)}^{2}\overset{-}{P}(1-\overset{-}{P})}{{\left(p_{1}-p_{2}\right)}^{2}}=34$$

$$\overset{-}{P}=\;\frac{\left(p_{1}+p_{2}\right)}{2}$$

$$Z_{1-\frac{\alpha }{2}}=1.96$$

z_1-β_=0.84

p_1_=0.45

p_2_=0.15

The study design and protocol of the study is shown in figure 1.

The instrument of this study consisted of two parts including a demographic questionnaire and the Pittsburgh Sleep Quality Index. The demographic questionnaire consisted of questions regarding participants’ demographic characteristics, and a single question about their sleep quality. We established the validity of the demographic questionnaire by using the content validity method. On the other hand, we evaluated the reliability of the questionnaire by calculating the intra-class correlation coefficient, which was equal to 0.86.

The second part of the instrument was the Pittsburgh Sleep Quality Index (PSQI).^25^ PSQI is a self-report questionnaire that assesses sleep quality and sleep problems during the previous month. It consisted of nineteen questions in seven domains including subjective sleep quality, sleep latency, sleep duration, habitual sleep efficiency, sleep disturbances, use of sleep medication, and daytime dysfunction. The possible answers to PSQI items are ‘Not during the past month,’ ‘Less than once a week,’ ‘Once or twice a week,’ and ‘Three or more times a week,’ which scored from 0 to 3, respectively. A mean score is calculated for each domain, which ranges from 0 to 3. Consequently, the total score of PSQI ranges from 0 to 21. According to the developers of PSQI, scores higher than 5 indicate poor sleep quality.^26^ The psychometric property of the Persian version of PSQI was evaluated and reported a specificity of 0.93 and a Chronbach’s alpha of 0.89 in Iran by Farrahi et al.^27^

Patients in the control group received the routine care of the study setting that included neither systematic training nor follow-up care. On the other hand, all the 34 patients in the experimental group were divided into four seven-person groups and one six-person group. Each group received the study intervention separately in two weeks (stages 1 and two),was followed up for two months, and immediately participated in post-test. The study intervention was a care plan designed based on the CCM. As mentioned earlier, CCM consisted of four stages. We went through these stages as follows.

1. Orientation: In this stage, we studied the patients’ medical records and recruited the eligible patients to the study. Then, in a 20–40-minute meeting, we explained the aim and process of the study to the recruited patients and their family members, invited them to actively participate in the study, and encouraged them to avoid ceasing their relationship with the researchers. Moreover, mutual expectations were also discussed in these meetings.
2. Sensitization: In this stage, we provided the study participants in the experimental group with information about the non-pharmacological management strategies of sleep problems including cognitive-behavioral therapy which in turn consisted of cognitive therapy, sleep hygiene education, and stress management and behavioral therapy. The educational materials regarding each of these strategies were as follows:
  - Cognitive therapy: We provided the study participants with information regarding DM, acute and chronic complications of DM, DM management, prevention of DM complications, the role of sleep in the management of DM, and different types of sleep problems.
  - Sleep hygiene education: We instructed the study participants about the effects of medications, sleeping environment (its light, noise, and temperature), and lifestyle habits (such as sleeping habits, exercise, and smoking) on sleep quality.
  - Behavioral therapy: The study participants were provided with information about the ways of establishing healthy sleeping habits, ways of increasing sleep duration, and techniques for muscle relaxation.
  - Stress management: We provided the study participants with information regarding effective management of stressful conditions as well as emotional and psychological strains.
  Each sub-group of patients in the experimental groups received these educational materials in four 60–90-minute sessions in two weeks. The sessions were organized according to the participants’ preferences and convenience. The cornerstone of CCM is maintaining the continuity of care. In other words, in CCM, establishing an effective long-term nurse-client relationship and mutual understanding of each other are matters of great importance. Accordingly, in the training sessions, we emphasized the importance of regular phone counseling and maintenance of care continuity. We also managed to refer patients who needed specialized services and trainings to clinical specialists. Moreover, we provided the participants with a booklet containing the above-mentioned information in a simplified, jargon-free language.
3. Control: In this stage, we tried to make regular contact with the participants to encourage them in making use of the provided information in their daily lives, to assess their problems, answer their questions, and provide them with counseling services. This stage lasted for two months.
4. Evaluation: in this stage, we again administered the PSQI to the study participants in both the experimental and the control groups; moreover, we assessed the patients in the experimental group in terms of the possibility of continuation and internalization of the educated behaviors.

In this study, we did not ask the participants to stop their tranquilizing or sedative medications. After the completion of the study and conducting the study post-test, we provided the study participants in the control group with a copy of the booklet containing the same educational materials provided to the participants in the experimental group. 

The gathered data were analyzed in SPSS (V. 20) using t-test and Chi-square statistics. In this study, the significance level was considered at 0.05. 

The Institutional Review Board and Ethics Committee of Gonabad University of Medical Sciences approved the study. Moreover, the Iranian Registry of Clinical Trials approved and registered this study (the registration number was IRCT201202269140N1). As mentioned earlier, we explained the aim and the process of the study to the study participants and guaranteed the confidentiality of their personal information. We also ensured them that both participation in and withdrawal from the study were voluntary. Finally, a written informed consent was obtained from the entire study participants. 

## Results

The study findings revealed that the mean (M) and standard deviation (SD) of the participants’ age were 57.82 and 9.35, respectively. Most of the participants (68.0%) were female patients. Regarding their educational status, 82.0% held high school diploma and 73.5% of female patients were housewives. The M and SD of Fast Blood Sugar (FBS) in intervention group was152.52±53.87 and in the control group it was155.38±54.98; according to independent-samples T-Test, there was no significant difference between the two groups before (P=0.820).Moreover, according to the study participants’ self-evaluation of sleep quality, 58.5% of them had sleep shortage, 12.5% had insomnia, 12.0% overslept, 11.0% had disturbed sleep, and 6% reported more than one sleep problem. The results of the independent-samples t and the Chi-square tests revealed that the study groups did not differ significantly in terms of age (P=0.12), gender (P=0.23), education (P=0.33) and employment status (0.73), and self-evaluated sleep quality (P=0.66) as well as based on the total score of PSQI, patients in both groups suffered from sleep problems (total score ≥5). On the other hand, the results revealed that after the study, the study groups did not differ significantly in terms of all seven domains of PSQI (Table 1). Moreover, the results of Chi-square test indicated that after the study, there was no statistically significant difference between the study groups regarding sleep quality (P=0.62; Table 2).

**Table 1:** Between- and within-group comparison of PSQI and its seven domains before and after the study

**Study variables**	** Time** **Group**	**Before**	**After**	P value*
		**mean±SD**	**mean±SD**	
Subjective sleep quality	Intervention	1.32±0.91	0.97±0.67	0.063
	Control	1.21±0.84	1.00±0.73	0.314
	P value †	0.583	0.684	——
Sleep latency	Intervention	1.077±1.58	1.05±0.98	0.034
	Control	1.14±0.95	1.14±1.04	1.000
	P value †	0.079	0.721	——
Sleep duration	Intervention	2.05±0.950	0.91±1.05	< 0.001
	Control	1.79±1.00	1.00±1.01	<0.001
	P value †	0.270	0.726	——
Habitual sleep efficiency	Intervention	1.35±1.27	0.29±0.75	< 0.001
	Control	0.82±1.11	0.38±0,88	0.041
	P value †	0.073	0.661	——
Sleep disturbances	Intervention	1.42±0.56	1.15±0.61	0.037
	Control	1.44±0.61	1.41±0.78	0.822
	P value †	1.00	0.137	——
Use of sleeping medication	Intervention	1.09±0.64	0.23±0.78	0.014
	Control	0.44±1.07	0.61±1.23	0.488
	P value †	0.438	0.131	——
Daytime dysfunction	Intervention	0.78±0.52	0.20±0.73	0.117
	Control	0.44±0.78	0-41±0.85	0.851
	P value †	0.645	0.290	——
PSQI total score	Intervention	9.05±2.95	4.79±3.68	<0.001
	Control	7.41±2.37	5.97±4.23	0.079
	P value †	0.014	0.226	——

*Paired- sample t test; † Independent-samples t test

**Table 2:** Between-group comparison of sleep quality after the study

**Group**	**Good Sleeper**		**Poor Sleeper**		**Chi-square test ** **P value **
	**No**	**%**	**No **	**%**	
Intervention	18	53	16	47	0.628
Control	16	47	18	53	
Total	34	100	34	100	

On the other hand, the results of the paired-samples t-test showed that after the study, the experimental group’s mean scores of sleep latency (P=0.034), sleep duration (P<0.001), habitual sleep efficiency (P<0.001), sleep disturbances (P=0.037), and use of sleeping medication (P=0.014) domains decreased significantly. Also, the total mean score of PSQI reduced from 9.05±2.95 to 4.79±3.68 that is a normal range (Table 1). However, in the control group, only the mean scores of the sleep duration (P<0.001) and sleep efficiency (P=0.041) domains decreased significantly after the study (see Table 1).

## Discussion

The aim of this study was to investigate the effects of continuous care model on sleep quality and disease control in patients with type 2 diabetes. The study findings revealed that the difference between the study groups in terms of sleep quality was not statistically significant before and after the study. However, the within-group comparisons showed that the experimental groups’ mean of PSQI and its sleep latency, sleep duration, habitual sleep efficiency, sleep disturbances, and use of sleeping medication domains decreased significantly after the study. In other words, after the study, patients in the experimental group reported having shorter sleep latency, longer sleep period, and greater satisfaction with the quality of their sleep. Moreover, in the experimental group, the mean of PSQI total score decreased to 4.79 after the study. In other words, after the study, it was within the range of superior sleep quality. The sleep quality of patients receiving hemodialysis improved significantly after the implementation of CCM.^20^ Another researcher also found that after the implementation of CCM, the sleep quality of chemical warfare victims suffering from obstructive bronchiolitis improved significantly in three domains of PSQI including subjective sleep quality, sleep disturbances, and use of sleep medication. However, after their study, the mean of PSQI total score decreased from 14.36±4.85 to 11.86±5.3, which was not within the range of superior sleep quality.^26^ Sivertsen et al. also found that compared to medication therapy, cognitive behavioral therapies including sleep hygiene education, relaxation, stress management, and cognitive therapy led to more increase in the efficiency and duration of sleep and more decrease in the length of nighttime insomnia among older adults.^14^

There is a great controversy about the length of non-pharmacological therapy prior to the onset of pharmacological management of sleep problems. This greatly depends on the severity of sleep problems, the severity of underlying diseases, and patient’s preferences. However, previous studies revealed that cognitive-behavioral therapies administered concurrently with a 7–8-week course of drug therapy produce the most beneficial results.^15^ The length of cognitive-behavioral therapy in our study was also two months. However, other studies supported longer periods.^14^ It is noteworthy that the difference between our findings and the findings of other studies is probably because that the focus of other studies has been on the management of specific sleep problems while the main problem of our participants was DM and the focus of our study was on managing sleep problems in general. Moreover, the intervention of other studies included cognitive-behavioral therapy in combination with the manipulation of dietary and exercise regimen. However, we did not manipulate the patients’ treatment, diet, and exercise regimens; nonetheless, our CCM-based non-pharmacological intervention significantly improved the sleep quality of patients in the experimental group.

The main limitations of the current study were ourshort time follow up and lack of focus on a special type of sleep problems. Our participants reported that they suffered from different types of sleep problems including sleep loss, insomnia, and disturbed sleep. However, PSQI is a general questionnaire that generally assesses the presence or absence of sleep disorders of any type. Accordingly, the use of specific sleep quality assessment questionnaires and investigation of the effects of CCM on specific types of sleep problems are recommended. Moreover, given the probable effects of uncontrolled blood sugar and sleep problems on each other, investigating the effects of non-pharmacological management of sleep problems in combination with blood sugar controlling modalities (such as the manipulation of dietary and exercise regimens) on the management of DM and the DM-related sleep problems is also recommended. 

## Conclusion

Although much has remained to be done, our work shows non-pharmacological sleep management intervention based on CCM helps the healthcare to reduce the mean score of sleep disorder in the experimental group. Therefore, providers manage diabetic patients’ sleep problems and hence, can use this model to improve patients’ sleep quality. Considering the prevalence of DM worldwide, the importance of its complications, its chronic and complex nature, and the direct effects of sleep problems on glucose intolerance, sleep care in DM is a matter of great importance and deserves particular attention. 
